# Structural Catalytic Core in Subtilisin-like Proteins and Its Comparison to Trypsin-like Serine Proteases and Alpha/Beta-Hydrolases

**DOI:** 10.3390/ijms252211858

**Published:** 2024-11-05

**Authors:** Alexander I. Denesyuk, Konstantin Denessiouk, Mark S. Johnson, Vladimir N. Uversky

**Affiliations:** 1 Structural Bioinformatics Laboratory, Biochemistry, InFLAMES Research Flagship Center, Faculty of Science and Engineering, Åbo Akademi University, 20520 Turku, Finland; kdenessi@abo.fi (K.D.); mark.s.johnson@abo.fi (M.S.J.); 2 Department of Molecular Medicine and USF Health Byrd Alzheimer’s Research Institute, Morsani College of Medicine, University of South Florida, Tampa, FL 33612, USA

**Keywords:** subtilisin-like, alpha/beta-hydrolases, serine proteases, 3D structure, structural catalytic core, catalytic pentad

## Abstract

Subtilisin-like proteins are serine proteases that use two types of catalytic triads: Ser-His-Asp and Ser-Glu-Asp. Here, we investigate the two known families of subtilisin-like proteins, the subtilases (Ser-His-Asp triad) and the serine-carboxyl proteinases (Ser-Glu-Asp triad), and describe the local structural arrangements (cores) that govern the catalytic residues in these proteins. We show the separation of the cores into conserved structural zones, which can be repeatedly found in different structures, and compare the structural cores in subtilisin-like proteins with those in trypsin-like serine proteases and alpha/beta-hydrolases.

## 1. Introduction

A large majority of proteins from the superfamilies of trypsin-like serine proteases and alpha/beta-hydrolases are enzymes that function through the use of a catalytic triad [1,2]. According to the MEROPS database, enzymes of these two superfamilies belong to clan PA (mixed C/S/T catalytic type) serine peptidases and clan SC serine peptidases, respectively [3]. Despite differences in protein class and fold (SCOP database [4]), the proteins of both superfamilies show structural similarity in the arrangement of their active sites [5,6]. For example, both have an additional “fourth” conserved residue interacting with the catalytic triads and bound to the respective catalytic bases by weak hydrogen bonds. Derewenda (2023) recently wrote a comprehensive review on the role of weak hydrogen bonds in the structures of proteins and nucleic acids [7]. Independently, it was also reported that subtilisin-like superfamily proteins [8,9], which belong to clan SB serine peptidases [3], also have a similar 3D arrangement of four catalytic residues mentioned above [5,6], which we will refer to as the “catalytic tetrad” of serine proteases when specifically speaking about the three aforementioned superfamilies. As opposed to trypsin-like serine proteases, the alpha/beta-hydrolases have five key residues (the catalytic triad plus two) to carry out protein function, which we, therefore, call a “catalytic pentad” [10].

Earlier, we had described the structural catalytic core (SCC) in trypsin-like serine proteases and in alpha/beta-hydrolases [11,12]. The core description method was based on the assumption that similar arrangements of key amino acids, such as the catalytic triads, can be found in the active sites of unrelated proteins where these key amino acids (acid, base, and nucleophile in a catalytic triad) are positioned in specific places with respect to each other. Therefore, it should not be surprising to find other supporting or interacting amino acids that could also be placed in the equivalent positions in space (structurally conserved). This is because they interact with the same groups in a similar way and together create similar local structural environments that we call “structural catalytic cores” (SCCs) in functionally unrelated proteins.

Furthermore, these overall similar structural environments, the SCCs, in the active sites were divided into “bricks”, i.e., smaller structural units of several amino acids in size, which usually contain one or several key amino acids, their supporting amino acids, and, importantly, they are interlocked by bonds. Thus, they can be considered as pseudo-independent, closed, small structural units that can be repeatedly found in the active sites of different proteins. These small, conserved, and closed structural brick units, which usually reflect the environments around one or several key functional groups, we call “zones”, giving rise to names like the “catalytic acid zone” or the “acid-base zone”, and so on. In order for a structural arrangement to be a zone, it should be a small unit of several amino acids in size; contain a functional element; be considered a structurally independent, closed or sometimes circular substructure, i.e., it is interlocked by bonds and hydrophobic interactions; and it could be found in several different proteins. Each zone incorporates a segment of the SCC and governs its respective element of protein functional machinery through a network of conserved hydrogen bonds and other interactions.

Here, we aim to describe the SCCs in subtilisin-like superfamily proteins and compare them to SCCs of trypsin-like serine proteases and alpha/beta-hydrolases, whose SCCs were found to be different from the subtilisin-like superfamily proteins despite the similarity in the 3D arrangement of their catalytic triads.

## 2. Results and Discussion

As described in the Introduction, various clans of serine proteases and alpha/beta-hydrolases have sets of four or five key residues called catalytic tetrads or catalytic pentads to carry out their function. These sets of key residues are usually incorporated into their respective zones, which taken together constitute the structural catalytic core (SCC). The zones can be used as convenient tools to compare the active sites of different “catalytic triad” enzymes with other proteins that have the same fold but serve different functions. Here, we begin with the previously observed fact that subtilisin-like proteins have a catalytic tetrad, as seen in trypsin-like serine proteases and alpha/beta-hydrolases, and proceed with the identification of the subtilisin SCC, making an inventory of key catalytic residues and the other elements of the SCC. We compare the respective SCCs in subtilisin-like proteins, trypsin-like serine proteases, and alpha/beta-hydrolases to determine how similar or different catalytic cores are among these three enzyme superfamilies.

### 2.1. Creating a Dataset of the Subtilisin-like Superfamily Proteins

The Protein Data Bank (PDB [13]) was used to retrieve the representative structures of the subtilisin-like superfamily proteins. In the SCOP database, the subtilisin-like superfamily includes two families, the subtilases and the serine-carboxyl proteinases (SCPs), with 3D structures of 42 and 3 different proteins, respectively [4]. For each of the 45 proteins from SCOP, one representative 3D structure with the highest resolution has been chosen (Table S1). Additionally, outside of the SCOP database, eight different structures, seven subtilases and one SCP, were selected to be included within the subtilisin-like superfamily, thus totaling 53 representative PDB ID entries (Table S1). Additional members were identified using the InterPro database (https://www.ebi.ac.uk/interpro/entry/InterPro/IPR000209/; accessed on 15 August 2024).

### 2.2. SCC in Subtilisin Savinase (Representative Structure of the Subtilases Family; Subtilisin-like Superfamily)

#### 2.2.1. Five Key Functional Amino Acids in Subtilisin Savinase

Based on the criteria above, the structure of the subtilisin savinase (PDB ID: 1GCI; R = 0.78 Å) [14] can be accepted as the representative structure of the overall subtilisin-like superfamily. Unlike trypsin-like serine proteases but similar to alpha/beta-hydrolases, subtilisin savinase displays a catalytic pentad of key functional residues. Three of the five residues—Asp32 (Acid), His64 (Base), and Ser221 (Nucleophile (Nuc))—are the actual catalytic triad (Table 1 and Table S1). The fourth residue, Ser125, forms a weak C–H·O hydrogen bond between its carbonyl oxygen and the side chain group of the catalytic base. We will refer to this amino acid as the “CHO”. Finally, the last amino acid of the catalytic pentad is Asn155 (Oxy). The backbone amide of Ser221 forms the oxyanion hole, which does not require introduction [15].

#### 2.2.2. AcidBaseCHO Zone

**Figure 1:**
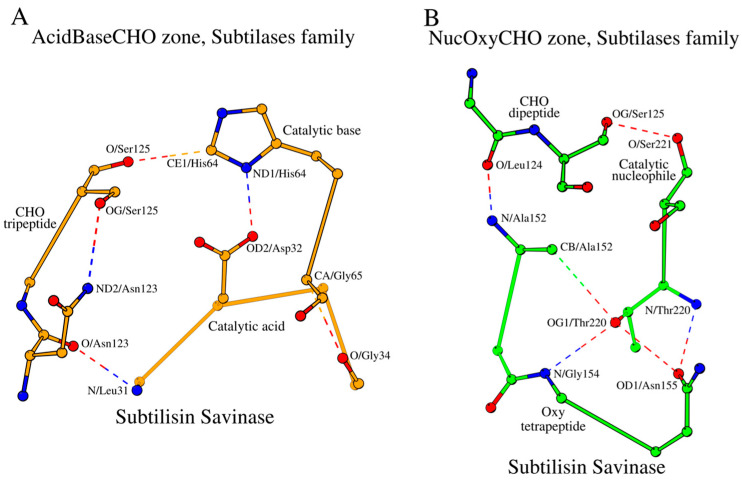
Two zones in the active site of subtilisin savinase (PDB ID: 1GCI), as a representative member of the subtilases family. (**A**) AcidBaseCHO zone (orange color) and (**B**) NucOxyCHO zone (green color). The dashed lines show long- and short-range hydrogen bonds (canonical and weak) between the bordering amino acid fragments of the primary structure of the protein, thus determining the cyclic nature and composition of the residues of each zone separately.

Let us consider the local substructures, which govern the five key amino acids described above. Three continuous fragments of the subtilisin savinase—the tetrapeptide Leu31-Gly34, which includes the catalytic acid Asp32; the dipeptide His64-Gly65, which includes the catalytic base His64; and the tripeptide Asn123-Ser125, which includes the CHO residue Ser125—form a closed structural formation locked by hydrogen bonds, which we will refer to as the “AcidBaseCHO zone” (Figure 1A; Table 2). For convenience purposes, we will refer to amino acids of the zone by a name and a number. For example, the first amino acid from the segment containing the catalytic acid is referenced as “Acid1”, and so on. In addition to the contacts that lock the AcidBaseCHO zone, Figure 1A and Table 2 also show contacts between catalytic residues, as well as the other internal stabilizing contacts of the segments of the zone. The cut-off for the distance of the canonical hydrogen bonds was ≤3.2 Å. With the exception of two contacts in Table 2 (SCP family), all other canonical hydrogen bonds satisfy this criterion. A slight increase in the distance between the analyzed atoms is observed in the case of a contact involving glutamic acid as a catalytic base. In the overall context of the presented data, these deviations do not affect our structural conclusions. The cut-off for distances of the C–H·O weak hydrogen bonds was ≤4.0 (3.0) Å with an angle ≥130° [7]. Only one contact in Table 2 slightly exceeds these criteria. This contact is observed in the proprotein convertase subtilisin/kexin type 9. As with canonical hydrogen bonds, this structural deviation does not change the overall picture.

#### 2.2.3. NucOxyCHO Zone

Two of the five key functional amino acids of subtilisin, the catalytic nucleophile (Nuc; Ser221 in 1GCI) and the oxyanion hole (Oxy; Asn155 in 1GCI), fall out of the AcidBaseCHO zone. They form their own closed substructure, the NucOxyCHO zone (Figure 1B; Table 3). The NucOxyCHO zone is formed through the interlocking of the ends of the Nuc dipeptide (Thr220-Ser221), the Oxy tetrapeptide (Ala152-Asn155), and the Leu124-Ser125 segment of the CHO tripeptide (Figure 1B). The OG1 atom of Thr220 seems to be the center of coordination of the “NucOxy” sub-zone due to its contacts with atom CB/Ala152, N/Gly154, and OD1/Asn155 (Figure 1B; Table 3). Only one contact in Table 3 slightly exceeds the cut-off for distances of the C–H·O weak hydrogen bonds. Once again, this contact is observed in the proprotein convertase subtilisin/kexin type 9.

#### 2.2.4. SCC as a Structural Association of AcidBaseCHO and NucOxyCHO Zones

**Figure 2:**
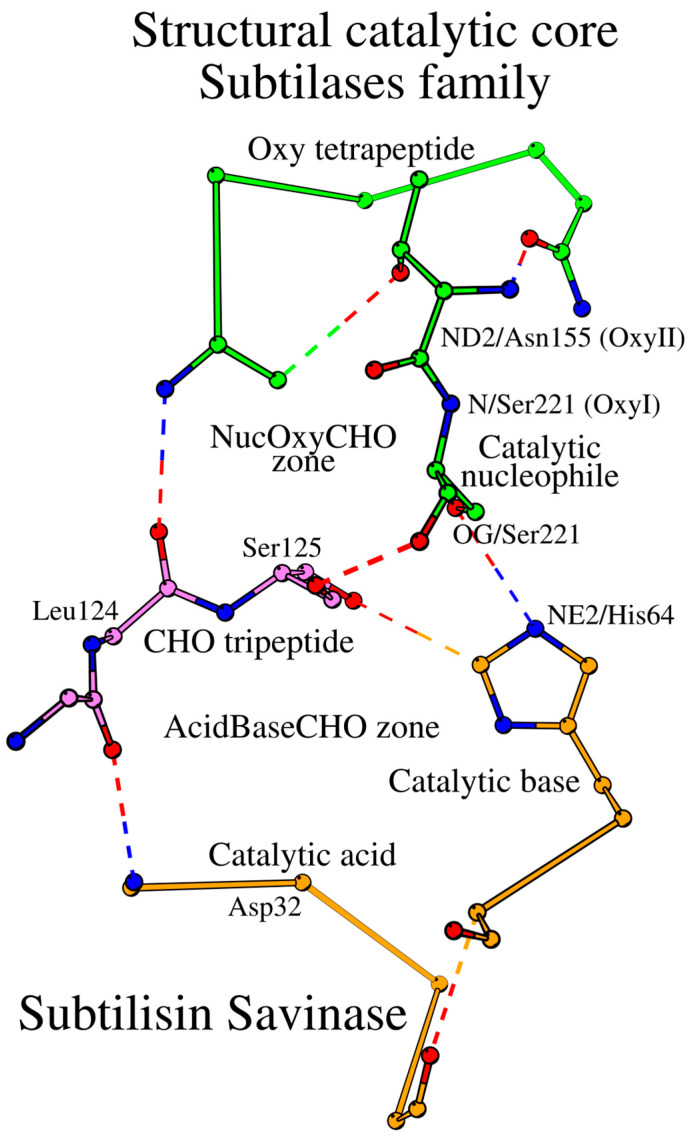
Structural catalytic core (SCC) of subtilisin savinase. CHO tripeptide (violet color) is a key common structural element of the two catalytic zones, colored orange and green.

**Figure 3:**
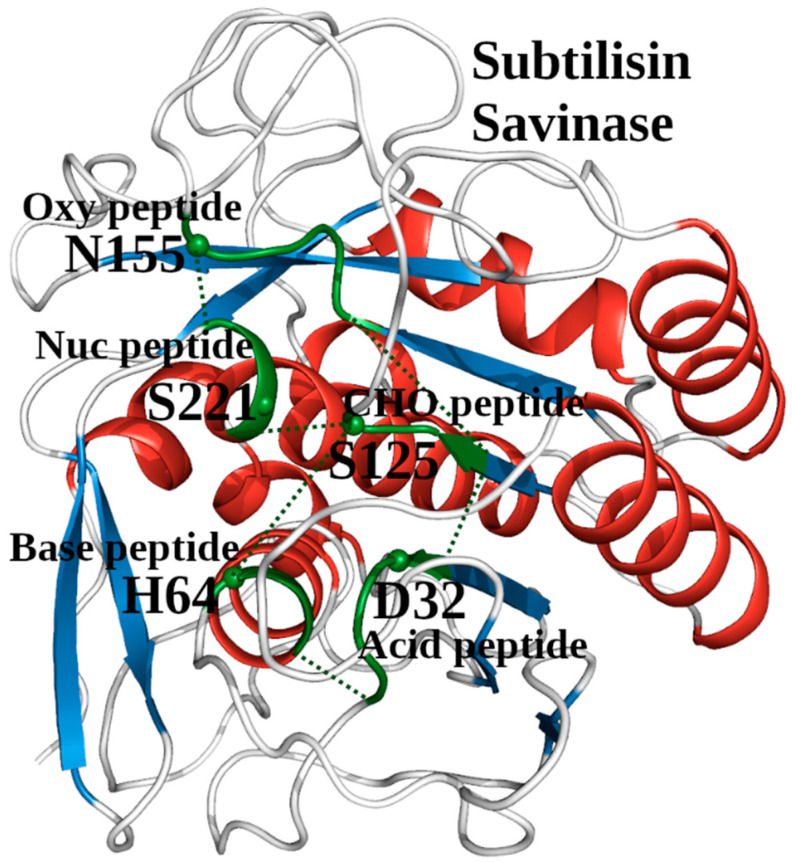
The 3D structure of the active site in subtilisin savinase (PDB ID: 1GCI). Five short amino acid segments show the location of the structural catalytic core (SCC). It consists of the acid, base, CHO, Oxy, and Nuc peptides (green color). The green dotted lines between the end residues of these five functional segments indicate the contacts that form the AcidBaseCHO and NucOxyCHO zones, respectively. The discussed catalytic amino acids are highlighted and labeled.

As demonstrated above, the catalytic acid/catalytic base and the catalytic nucleophile/oxyanion hole environments are two different localized environments, referred to as the AcidBaseCHO and the NucOxyCHO zones, respectively. The two zones are linked by their common element, the Leu124-Ser125 dipeptide of the CHO tripeptide (Figure 2). Taken together, the two zones constitute the SCC of the subtilisin savinase, which includes 15 amino acids from 5 different peptides (Table 1). The location of the SCC within the 3D structure of savinase is shown in Figure 3.

### 2.3. SCC of the Other Subtilases (Subtilisin-like Superfamily): Variations in the CHO Peptide

After examining the subtilisin savinase, the remaining representative structures from the subtilase family of subtilisin-like proteins were similarly analyzed for the SCCs formed from fifteen residues in five peptides and incorporating five key functional amino acids. The results are summarized in Table S1 in the form of a structural alignment. All structural superpositions were done using the Dali server [21]. As shown above, the CHO peptide belongs to both the AcidBaseCHO and NucOxyCHO zones and joins them together into the SCC. All subtilases have one of three types of CHO peptides: CHO1 = Asn (the Asn group; 33 structures in Table S1); CHO1 = Ser/Thr (the Ser/Thr group; 14 structures in Table S1); and two exceptions in the proprotein convertase subtilisin/kexin type 9 and thiazoline oxidase/subtilisin-like protease, where CHO1 is not Asn, Ser, or Thr, and the polar CHO1-CHO3 contact is missing (the Xaa group in Table S1). In the proteins of the Asn group and the Ser/Thr group, CHO3 = Ser. The change from Asn to Ser at the CHO1 position in group 2 results in the inclusion of the water molecule, HOH_I_, as an intermediate link between CHO1 and CHO3 (Table 2 and Table S1). The hydrophobic CHO1-CHO3 contact (Leu286-Pro288 in proprotein convertase subtilisin/kexin type 9 of the Xaa group) does not contain a HOH_I_ molecule between the two amino acids of the contact, as is the case in the Asn group (Table 2). Similarly, the intermediate HOH_I_ water molecule is absent in contact His681-Ala683 in the thiazoline oxidase/subtilisin-like protease of the Xaa group. The absence of a HOH_I_ water molecule in both proteins is likely due to the large sizes of the side chain groups of residues Pro288 and His681.

Due to the important role of the CHO peptide in the formation of the native functional contact between the catalytic nucleophile and the catalytic base, it can be speculated that there should be three structurally different active sites in the subtilase family. The importance of the difference in amino acids in the CHO1 and CHO2 positions of the CHO tripeptide, as well as the presence or absence of the HOH_I_ water molecule between them, for the structural organization of the AcidBaseCHO zone in the subtilase family will be further described in detail in Section 2.5.

### 2.4. SCC in Serine-Carboxyl Proteinases (SCP Family; Subtilisin-like Superfamily)

**Figure 4:**
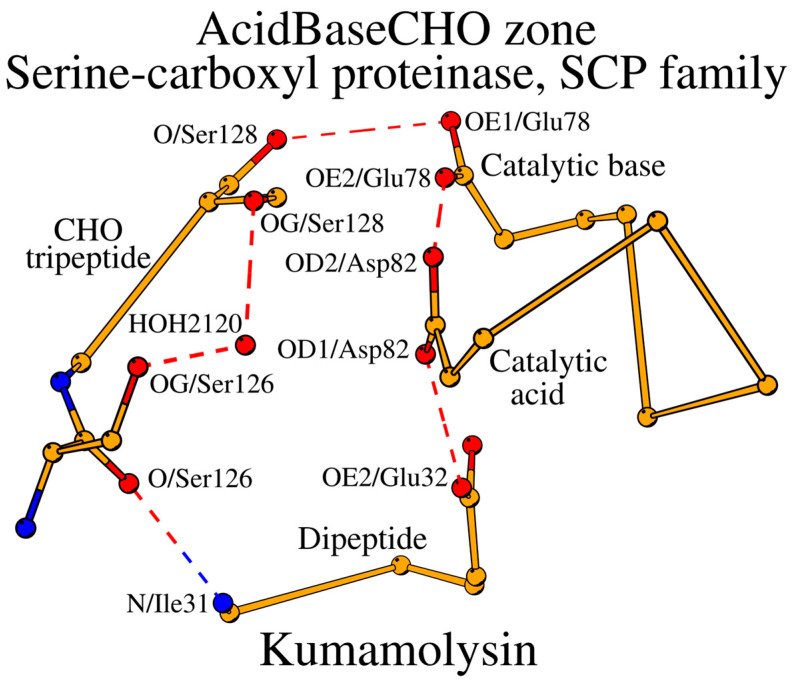
AcidBaseCHO zone (orange color) of kumamolysin (PDB ID: 1GT9). Water HOH_I_ (HOH_2120_) is shown to stabilize the conformation of the CHO tripeptide.

The SCP family of subtilisin-like proteins includes only a few known representative structures (Table S1). Similar to the subtilases, the SCP enzymes can be divided into the Asn and Ser/Thr groups according to the structure of the CHO peptide. Kumamolysin, a member of the Ser/Thr group, can be selected as the representative structure of the SCP family (Table 1). Both subtilases and the SCP enzymes have five similarly placed key catalytic residues: the catalytic triad plus CHO plus Oxy. However, the main difference between subtilases and the SCP family is in the construction of their SCCs. The key catalytic acid in the SCP occupies the typical location in the structure, but in sequence, it moves to the C-terminal end of the peptide, which contains the catalytic base, i.e., changing from the peptide “acid” location in subtilases to the peptide “base” location (Table 1; Figure 4). Additionally, the catalytic base in the SCP is always Glu instead of His. As a result, the “acid” tetrapeptide turns into a dipeptide in the SCP, and the “base” dipeptide becomes a chimeric “BaseAcid” pentapeptide with the following structure: catalytic base (BaseAcid1)–X2–X3–X4–catalytic acid (BaseAcid5). The incorporation of both the catalytic base and acid in the same peptide while maintaining their functional relation is performed by the α-helical conformation of the BaseAcid peptide. In a canonical α-helix, the first residue (BaseAcid1; catalytic base) and the fifth residue (BaseAcid5; catalytic acid) are usually connected by a canonical helix-forming hydrogen bond; at the same time, they serve the enzymatic function as part of the catalytic triad. Thus, taken together, the subtilisin-like proteins can be divided into two major groups, where the catalytic acid occupies its own structural segment within the SCC (“Acid” in Figure 3 and Table 1) or the catalytic acid is placed within the same structural segment as the catalytic base (“Base” in Table 1). A similar division had been also observed in alpha/beta-hydrolases [22]. Moreover, the movement of catalytic residues from one structural element to another with the preservation of the spatial arrangement of side chains is not unusual; for example, this is observed in the PD-(D/E)xK phosphodiesterase superfamily of proteins [23].

**Figure 5:**
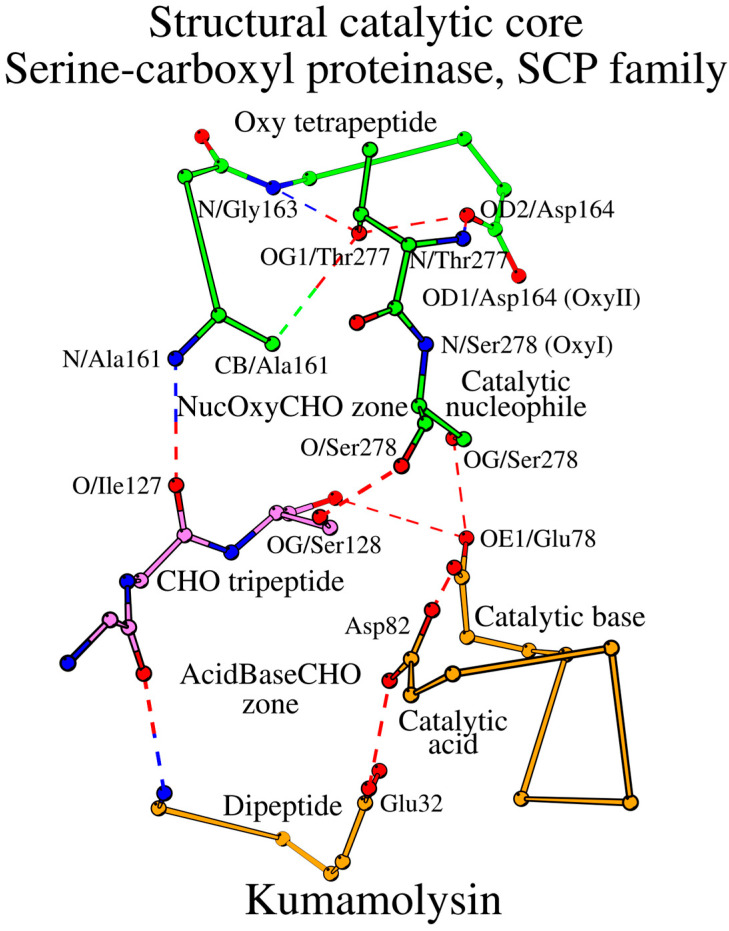
Structural catalytic core (SCC) of kumamolysin. CHO tripeptide (violet color) is a key common structural element of the two catalytic zones, colored orange and green.

Due to the structural rearrangement, the number of amino acids forming the SCC in the SCP family is 16. Additional changes include the Oxy4 residue transition Asn → Asp, and the presence of the water-mediator at the CHO1-CHO3 contact (Figure 4, Table 2). The entire SCC of the SCP family of subtilisin-like proteins is shown in Figure 5 and described in Table 3.

### 2.5. Invariant Water Molecule of the AcidBaseCHO Zone in the Subtilisin-like Superfamily Proteases

**Figure 6:**
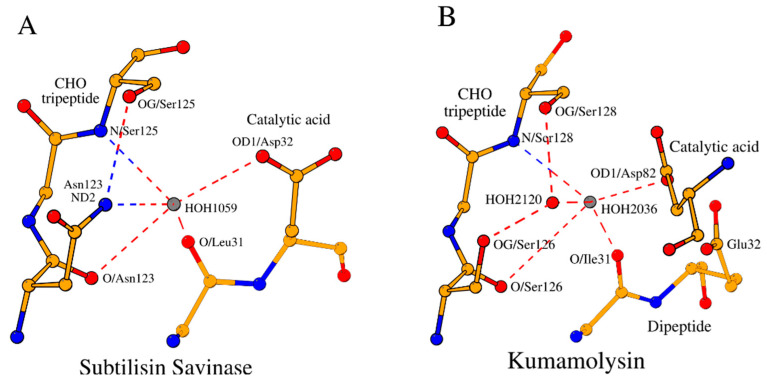
Invariant water HOH_II_ (grey color) in the subtilisin-like superfamily proteases. (**A**) Water HOH_1059_ of the AcidBaseCHO zone in the subtilisin savinase and (**B**) water HOH_2036_ (and relation to HOH_2120_ (HOH_I_, red color)) of the AcidBaseCHO zone in the kumamolysin.

As shown in Section 2.2.3, in subtilases, the OG1 atom of Thr220 seems to be the center of coordination of the NucOxyCHO zone due to its contacts with CB/Ala152, N/Gly154, and OD1/Asn155 (Figure 1B). The conserved structural water molecules play a somewhat similar coordinating role in the AcidBaseCHO zones in both the subtilases and SCPs (Figure 6A,B; Table 1, Table 4 and Table S1).

In subtilases, a water molecule (HOH_II_ in Table 4) coordinates the location of the catalytic acid with respect to the functionally important CHO3 residue (Figure 6A). Of the 53 analyzed structures, HOH_II_ water was not identified only in two cases: in the intracellular serine protease (PDB ID: 7Y6M; Table S1), likely due to the low resolution of the structure, and in the proprotein convertase subtilisin/kexin type 9 (PDB ID: 6U26; Table 4 and Table S1), due to proline at the CHO3 position. With the exception of two contacts (the SCP family), all other canonical hydrogen bonds satisfy the ≤3.2 Å criterion in Table 4. In the overall context of the presented data, these deviations do not affect our structural conclusions.

Above, we described that based on the composition of the CHO peptide, the subtilisin-like enzymes belong to either the Asn group or the Ser/Thr group. The two groups differ by the presence or absence of the second coordinating water molecule, HOH_I_, in the structure of the AcidBaseCHO zone that separates the two groups (Table S1).

### 2.6. Comparison of Subtilisin-like Enzymes and Alpha/Beta-Hydrolases: Catalytic Pentads

**Figure 7:**
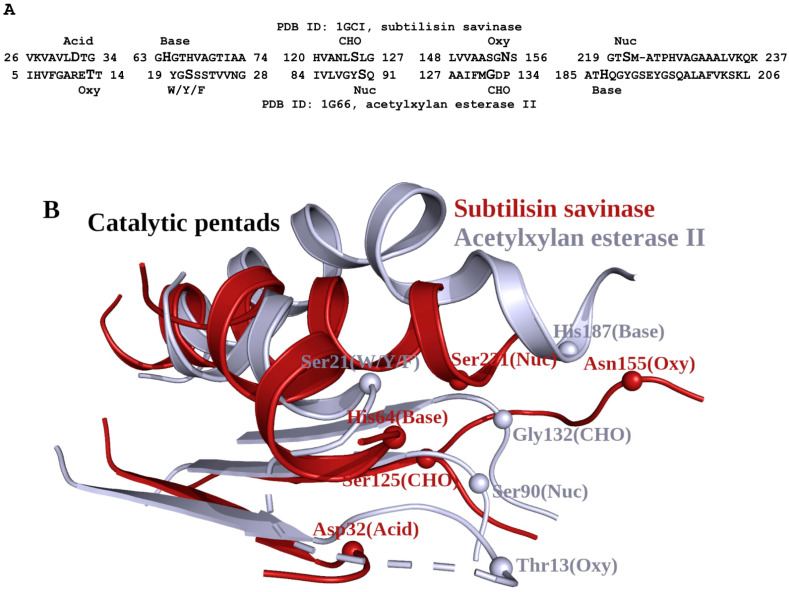
Catalytic pentads in subtilisin and acetylxylan esterase II. (**A**) Structural sequence alignment of these two enzymes. Five catalytically important residues in the sequence of both enzymes are shown in a larger font. (**B**) 3D superposition of the two alpha-helixes and three beta-strands in the subtilisin (dark red color) and acetylxylan esterase II (grey color). The positions, names, and functional roles of five catalytically important amino acids are shown.

Both subtilisin-like superfamily enzymes and alpha/beta-hydrolases share a central parallel β-sheet of 7 strands [4], and the pairwise superposition of the subtilisin savinase (PDB ID: 1GCI) and acetylxylan esterase II (PDB ID 1G66, [24]) using the Dali server [21] shows a z-score of 2.9 and an RMSD of 3.7 Å over 108 residues. The resulting structural alignment between subtilisin and acetylxylan esterase II, aligned segments in Figure 7A, includes five functionally important residues together with the adjacent secondary structure fragments: two α-helixes and three β-strands. From the alignment, it is evident that few identical amino acids are aligned; nevertheless, the five catalytic residues are located at the same termini of the same fragments of the secondary structure, albeit not in identical positions, but forming functional pentads at the same locations of the overall fold (Figure 7B). Note that in Figure 7B, the catalytic acid-containing fragment of acetylxylan esterase II is shown as W/Y/F, as described earlier in [25].

## 3. Materials and Methods

The SCOP classification database [4] and the Protein Data Bank (PDB, http://www.rcsb.org/; 15 August 2024 [13]) were used to retrieve 53 representative structures of proteins from the subtilisin-like superfamily (SCOP ID: 3000226). Detailed structural information from the above set of PDB files is given in Section 2.1.

Structure visualization and structural analysis of interactions between amino acids in proteins (hydrogen bonds, hydrophobic, other types of weak interactions) was conducted using Maestro (Schrödinger Release 2023-1: Schrödinger, LLC, New York, NY, USA, 2021; http://www.schrodinger.com/ release 15 August 2024) and the software to determine ligand-protein contacts (LPCs) and between structural units (CSUs) [26].

Pairwise structural superpositions were done using the Dali server (http://ekhidna2.biocenter.helsinki.fi/dali/; 15 August 2024) [21]. Weak hydrogen bonds were identified based on geometrical criteria [7]. The π-π stacking interactions and other interactions were analyzed using the Residue Interaction Network Generator (RING, https://ring.biocomputingup.it/; 15 August 2024) [20]. Figures were drawn with MOLSCRIPT [27] and PyMOL molecular graphics system (https://pymol.org/; accessed on 25 October 2024).

## 4. Conclusions

In this study, we have described a structural scaffold incorporating catalytic residues—the structural catalytic core (SCC)—in the subtilisin-like superfamily of enzymes. We showed that the SCC is roughly divided into two halves, which are two structurally conserved and locally interconnected structural organizations or zones, the AcidBaseCHO and the NucOxyCHO zone. The AcidBaseCHO zone governs the positioning of the catalytic acid and base, and the NucOxyCHO zone governs the positioning of the catalytic nucleophile and the oxyanion hole. The two zones are connected by the CHO peptide, which can only be of two types, dividing subtilisin-like enzymes into two groups: the Asn group and the Ser/Thr group. The AcidBaseCHO zone incorporates structurally conserved water molecules for the coordination of the catalytic acid, which are the key elements separating the Asn (one water) and Ser/Thr (two water molecules) groups.

Although the two known families within the subtilisin-like superfamily—the family of subtilases and the family of serine-carboxyl proteinases (SCPs)—have a similar arrangement of the catalytic residues and the catalytic cores, they differ in the positioning of the catalytic acid along the sequence. In the SCP family, the catalytic acid does not reside in its own segment of the SCC as seen in subtilases, but “moves” to the same segment of SCC containing the catalytic base.

The comparisons reveal that the subtilisin-like proteins are more similar to the alpha/beta-hydrolases compared to the trypsin-like serine proteases. Similarly to the alpha/beta-hydrolases, subtilisin-like proteins have five key functional amino acids, which are positioned at the same general location of the supersecondary structure. Most importantly, in the alpha/beta-hydrolases, we see the same division into the Asn and Ser/Thr groups, together with the “walk” of the catalytic acid to the structural segment of the catalytic base [22].

## Figures and Tables

**Table 1:** Structural alignment of five peptides ^a^ forming the SCC in 5 representative structures of the subtilisin-like superfamily of proteases.

**N**	**Protein**	**PDB ID**	**Acid**	**Base**	**CHO**	**Oxy**	**Nuc**	**HOH ^b^**	**Ref.**
Family: subtilases									
Asn group									
1	Subtilisin savinase	1GCI_A	^31^L**D**TG^34^	^64^**H**G^65^	^123^NL**S**^125^	^152^ASG**N**^155^	^220^T**S**^221^	1059	[14]
Ser/Thr group									
2	Putative 36kDa protease	2IXT_A	^33^L**D**TG^36^	^71^**H**G^72^	^137^SM**S**^139^	^166^AAG**N**^169^	^249^T**S**^250^	2271 2078	[16]
Xaa group									
3	Proprotein convertase subtilisin/kexin type 9	6U26_B	^185^L**D**TS^188^	^226^**H**G^227^	^286^LL**P**^288^	^314^AAG**N**^317^	^385^T**S**^386^	N/A ^c^	[17]
**N**	**Protein**	**PDB ID**	**Dipeptide**	**BaseAcid**	**CHO**	**Oxy**	**Nuc**	**HOH**	**Ref.**
Family: serine-carboxyl proteinase, SCP ^d^									
Asn group									
4	Serine-carboxyl proteinase	1GA6_A	^33^IT--^34^	^80^**E**WDL**D**^84^	^131^NV**S**^133^	^167^SSG**D**^170^	^286^T**S**^287^	403	[18]
Ser/Thr group									
5	Kumamolysin	1GT9_2	^31^IE--^32^	^78^**E**VEL**D**^82^	^126^SI**S**^128^	^161^AAG**D**^164^	^277^T**S**^278^	2120 2036	[19]

^a^ Five main catalytic residues are shown in bold. ^b^ The “HOH” column shows the PDB numbers of the HOH_I_ and HOH_II_ water molecules. If only one number is given, it belongs to the HOH_II_ water molecule. If two numbers are given, the upper number refers to HOH_I_, and the lower number refers to HOH_II_. ^c^ N/A—Not Available. ^d^ The designation “--” is used only to emphasize the presence of dipeptide without a catalytically important amino acid in the SCP family compared to the acid tetrapeptide in the subtilases family.

**Table 2:** Amino acid contacts forming the AcidBaseCHO zones in 5 representative structures of the subtilisin-like superfamily of proteases.

**N**	**Protein**	**PDB ID**	**AcidBaseCHO Zone**			**Acid_2_-Base_1_**	**CHO_1_-CHO_3_** **CHO_1_-HOH_I_-CHO_3_^a^**
			**Acid_1_-CHO_1_**	**Acid_4_-Base_2_**	**Base_1_-CHO_3_**		
Family: subtilases							
Asn group							
1	Subtilisin savinase	1GCI_A	N/L_31_-O/N_123_ 2.9	O/G_34_-CA/G_65_ 3.2 (2.4) 139^O^	CE1/H_64_-O/S_125_ 3.2 (2.3) 140^O^	OD2/D_32_-ND1/H_64_ 2.6	ND2/N_123_-OG/S_125_ 3.0
Ser/Thr group							
2	Putative 36kDa protease	2IXT_A	N/L_33_-O/S_137_ 3.1	O/G_36_-CA/G_72_ 3.2 (2.3) 136^O^	CE1/H_71_-O/S_139_ 3.2 (2.3) 146^O^	OD1/D_34_-ND1/H_71_ 2.6	OG/S_137_-HOH_2271_ 2.6 HOH_2271_-OG/S_139_ 2.8
Xaa group							
3	Proprotein convertase subtilisin/kexin type 9	6U26_B	N/L_185_-O/L_286_ 3.2	O/S_188_-CA/G_227_ 4.2 (3.4) 137^O^	CE1/H_226_-O/P_288_ 3.6 (2.7) 149^O^	OD2/D_186_-ND1/H_226_ 2.6	CG/L_286_-CD/P_288_ 4.1 CG/L_286_-CG/P_288_ 4.2
**N**	**Protein**	**PDB ID**	**AcidBaseCHO Zone**			**BaseAcid_5_-BaseAcid_1_**	**CHO_1_-CHO_3_** **CHO_1_-HOH_I_-CHO_3_**
			**Dipeptide_1_-CHO_1_**	**Dipeptide_2_-BaseAcid_5_**	**BaseAsid_1_-CHO_3_**		
Family: serine-carboxyl proteinase, SCP							
Asn group							
4	Serine-carboxyl proteinase	1GA6_A	N/I_33_-O/N_131_ 3.0	CA/T_34_-π/W_81_ 3.8 ^b^ CE3/W_81_-OD1/D_84_ 3.8 (2.8) 151^O^	OE1/E_80_-O/S_133_ 3.5	OD2/D_84_-OE2/E_80_ 2.6	ND2/N_131_-OG/S_133_ 2.9
Ser/Thr group							
5	Kumamolysin	1GT9_2	N/I_31_-O/S_126_ 3.2	OE2/E_32_-OD1/D_82_ 2.7	OE1/E_78_-O/S_128_ 3.3	OD2/D_82_-OE2/E_78_ 2.6	OG/S_126_-HOH_2120_ 2.7 HOH_2120_-OG/S_128_ 2.8

^a^ Water HOH_I_ stabilizes the conformation of the CHO tripeptide. ^b^ The π-π stacking and similar contacts were analyzed using the Residue Interaction Network Generator (RING) [20].

**Table 3:** Amino acid contacts forming the NucOxyCHO zone and NucOxy sub-zone in 5 representative structures of the subtilisin-like superfamily of proteases.

N	Protein	PDB ID	NucOxyCHO Zone				Nuc_1_-Oxy_3_	Nuc_2_-Base_1_
			CHO_2_-Oxy_1_	CHO_3_-Nuc_2_	NucOxy Sub-Zone			
					Nuc_1_-Oxy_1_	Nuc_1_-Oxy_4_		
Family: subtilases								
Asn group								
1	Subtilisin savinase	1GCI_A	O/L_124_-N/A_152_ 3.1	OG/S_125_-O/S_221_ 2.8	OG1/T_220_-CB/A_152_ 3.5 (2.5) 172^O^	N/T_220_-OD1/N_155_ 2.9 OG1/T_220_-OD1/N_155_ 2.9	OG1/T_220_-N/G_154_ 3.0	OG/S_221_-NE2/H_64_ 3.1
Ser/Thr group								
2	Putative 36kDa protease	2IXT_A	O/M_138_-N/A_166_ 3.0	OG/S_139_-O/S_250_ 2.7	OG1/T_249_-CB/A_166_ 3.5 (2.4) 173^O^	N/T_249_-OD1/N_169_ 2.9 OG1/T_249_-OD1/N_169_ 2.8	OG1/T_249_-N/G_168_ 3.1	OG/S_250_-NE2/H_71_ 2.9
Xaa group								
3	Proprotein convertase subtilisin/kexin type 9	6U26_B	O/L_287_-N/A_314_ 3.2	CB/P_287_-O/S_386_ 4.4 (3.5) 140^O^	OG1/T_385_-CB/A_314_ 3.4 (2.3) 174^O^	N/T_385_-OD1/N_317_ 2.9 OG1/T_385_-OD1/N_317_ 2.7	OG1/T_385_-N/G_316_ 3.1	OG/S_386_-NE2/H_226_ 3.1
Family: serine-carboxyl proteinase, SCP								Nuc_2_-BaseAcid_1_
Asn group								
4	Serine-carboxyl proteinase	1GA6_A	O/V_132_-N/S_167_ 3.1	OG/S_133_-O/S_287_ 2.9	OG1/T_286_-CB/S_167_ 3.4 (2.3) 164^O^	N/T_286_-OD2/D_170_ 2.9 OG1/T_286_-OD2/D_170_ 2.9	OG1/T_286_-N/G_169_ 3.0	OG/S_287_-OE1/E_80_ 2.7
Ser/Thr group								
5	Kumamolysin	1GT9_2	O/I_127_-N/A_161_ 3.0	OG/S_128_-O/S_278_ 2.8	OG1/T_277_-CB/A_161_ 3.4 (2.3) 169^O^	N/T_277_-OD2/D_164_ 2.8 OG1/T_277_-OD2/D_164_ 2.9	OG1/T_277_-N/G_163_ 3.0	OG/S_278_-OE1/E_78_ 2.6

**Table 4:** Contacts of the conserved water molecules with residues of the AcidBaseCHO zones in 4 representative structures of the subtilisin-like superfamily of proteases.

**N**	**Protein**	**PDB ID**	**Acid_1_-HOH_II_**	**Acid_2_-HOH_II_**	**CHO_1_-HOH_II_**	**CHO_3_-HOH_II_**	**CHO_1_/HOH_I_-HOH_II_^a^**
Family: subtilases							
Asn group							
1	Subtilisin savinase	1GCI_A	O/L_31_-HOH_1059_ 2.8	OD1/D_32_-HOH_1059_ 2.7	O/N_123_-HOH_1059_ 3.0	N/S_125_-HOH_1059_ 2.8	ND2/N_123_-HOH_1059_ 2.9
Ser/Thr group							
2	Putative 36kDa protease	2IXT_A	O/L_33_-HOH_2078_ 2.8	OD2/D_34_-HOH_2078_ 2.7	O/S_137_-HOH_2078_ 3.1	N/S_139_-HOH_2078_ 2.8	HOH_2271_-HOH_2078_ 2.8
Xaa group							
3	Proprotein convertase subtilisin/kexin type 9	6U26_B	O/L_185_-CD/P_288_ 3.1 (2.2) 138^O^	OD1/D_186_-CG/P_288_ 3.0 (1.9) 170^O^	O/L_286_-CD/P_288_ 3.3 (2.3) 147^O^	N/A ^b^	N/A
**N**	**Protein**	**PDB ID**	**Dipeptide_1_-HOH_II_**	**BaseAcid_5_-HOH_II_**	**CHO_1_-HOH_II_**	**CHO_3_-HOH_II_**	**CHO_1_/HOH_I_-HOH_II_**
Family: serine-carboxyl proteinase, SCP							
Asn group							
4	Serine-carboxyl proteinase	1GA6_A	O/I_33_-HOH_403_ 2.8	OD1/D_84_-HOH_403_ 2.8	O/N_131_-HOH_403_ 3.4	N/S_133_-HOH_403_ 2.9	ND2/N_131_-HOH_403_ 3.1
Ser/Thr group							
5	Kumamolysin	1GT9_2	O/I_31_-HOH_2036_ 2.7	OD1/D_82_-HOH_2036_ 3.0	O/S_126_-HOH_2036_ 3.7	N/S_128_-HOH_2036_ 2.9	HOH_2120_-HOH_2036_ 2.9

^a^ Water HOH_I_ stabilizes the conformation of the CHO tripeptide. Water HOH_II_ is an invariant water in the subtilisin-like superfamily of proteases. ^b^ N/A—Not Available.

## Data Availability

All data supporting reported results can be found in the Supplementary Materials.

## Supplementary Materials

The following supporting information can be downloaded at https://www.mdpi.com/article/10.3390/ijms252211858/s1. References [14,16,17,18,19,28,29,30,31,32,33,34,35,36,37,38,39,40,41,42,43,44,45,46,47,48,49,50,51,52,53,54,55,56,57,58,59,60,61,62,63,64,65,66,67,68,69] are cited in the Supplementary Materials.
