# Supplementary material for: Structural Catalytic Core in Subtilisin-like Proteins and Its Comparison to Trypsin-like Serine Proteases and Alpha/Beta-Hydrolases
Source: Int J Mol Sci. 2024 Nov 5;25(22):11858. doi: 10.3390/ijms252211858 (PMC11593635; doi:10.3390/ijms252211858)
Supplement: Supplementary file 1 [file ijms-25-11858-s001.zip › ijms-3273062-supplementary.pdf]

Supplementary materials

Structural Catalytic Core in Subtilisin-Like Proteins and its Comparison to Trypsin-Like Serine Proteases and Alpha/Beta-Hydrolases.

Alexander I. Denesyuk[1], Konstantin Denessiouk[1], Mark S. Johnson[1], Vladimir N. Uversky[2]

[1] Structural Bioinformatics Laboratory, Biochemistry, InFLAMES Research Flagship Center, Faculty of Science and Engineering, Åbo Akademi University, Turku 20520, Finland

[2] Department of Molecular Medicine and USF Health Byrd Alzheimer's Research Institute, Morsani College of Medicine, University of South Florida, Tampa, FL 33612, USA

Table S1. Structural alignment of five peptides[a] and water molecules forming SCC in 53 representative structures of the subtilisin-like superfamily proteases.

N	Protein	PDB ID	Acid	Base	CHO	Oxy	Nuc	HOH [b]	Ref.
Family: Subtilases									
Asn group									
1	Subtilisin Savinase	1GCI_A	31 L**D**TG 34	64 **H**G 65	123 NL**S** 125	152 ASG**N** 155	220 T**S** 221	1059	[1]
2	Subtilisin BPN	1TO2_E	31 I**D**SG 34	64 **H**G 65	123 NM**S** 125	152 AAG**N** 155	220 T**S** 221	463	[2]
3	Subtilisin Carlsberg	1R0R_E	31 L**D**TG 34	64 **H**G 65	123 NM**S** 125	152 AAG**N** 155	220 T**S** 221	4003	[3]
4	Subtilisin E	1SCJ_A	31 I**D**SG 34	64 **H**G 65	123 NM**S** 125	152 AAG**N** 155	220 T**C** 221	408	[4]
5	Extracellular subtilisin-like serine proteinase	1SH7_A	36 I**D**TG 39	70 **H**G 71	126 NM**S** 128	154 AAG**N** 157	219 T**S** 220	1303	[5]
6	Subtilisin-like protein	4H6V_A	22 L**D**GP 25	58 **H**G 59	117 NI**S** 119	150 AAG**N** 153	217 T**S** 218	403	[6]
7	Keratinase	5WSL_A	38 I**D**TG 41	72 **H**G 73	128 NM**S** 130	156 AAG**N** 159	223 T**S** 224	426	[7]
8	Alkaline protease	3QTL_A	31 L**D**TG 34	63 **H**G 64	122 NM**S** 124	151 AAG**N** 154	219 T**S** 220	278	[8]
9	KerA (Fragment)	4GI3_A	31 L**D**TG 34	64 **H**G 65	123 NM**S** 125	152 AAG**N** 155	220 T**S** 221	473	[9]
10	M-protease	1WSD_A	31 L**D**TG 34	64 **H**G 65	123 NL**S** 125	152 ASG**N** 155	220 T**S** 221	289	[10]
11	Acidic extracellular subtilisin-like protease AprV2	3LPA_A	40 V**D**TG 43	105 **H**G 106	174 NM**S** 176	204 AAG**N** 207	276 T**S** 277	367	[11]
12	N-terminal cyanobactin protease	4H6W_A	25 L**D**GI 28	61 **H**G 62	120 NV**S** 122	153 ATG**N** 156	220 T**S** 221	403	[6]
13	Serine protease (Fragment)	3F7O_A	40 I**D**TG 43	72 **H**G 73	133 NM**S** 135	161 AAG**N** 164	226 **TS** 227	314	[12]
14	Subtilisin-like protease 1	4LVN_A	371 I**D**SG 374	428 **H**G 429	488 NG**S** 490	517 SAS**N** 520	605 T**S** 606	802	[13]
15	Fervidolysin	1R6V_A	169 V**D**TG 172	208 **A**G 209	273 NH**S** 275	302 SAG**N** 305	388 T**S** 389	717	[14]
16	Subtilisin	1MEE_A	31 I**D**SG 34	64 **H**G 65	123 NM**S** 125	152 AAG**N** 155	220 T**S** 221	452	[15]
17	Thermophilic serine proteinase	1DBI_A	38 I**D**TG 41	72 **H**G 73	132 NL**S** 134	161 AAG**N** 164	225 T**S** 226	444	[16]
18	Subtilisin DY	1BH6_A	31 I**D**TG 34	64 **H**G 65	123 NM**S** 125	152 AAG**N** 155	220 T**S** 221	534	[17]
19	Subtilisin BL	1ST3_A	31 L**D**TG 34	62 **H**G 63	121 NL**S** 123	150 ASG**N** 153	214 T**S** 215	283	[18]
20	Enzyme subtilase SubTY from *Bacillus* sp. TY145	5FFN_A	34 L**D**TG 37	72 **H**G 73	138 NM**S** 140	167 AAG**N** 170	250 T**S** 251	553	[19]
21	Proteinase K	2B6N_A	38 I**D**TG 41	69 **H**G 70	130 NM**S** 132	158 AAG**N** 161	223 T**S** 224	405	[20]
22	Microbial serine proteinases	2GKO_A	33 L**D**TG 36	71 **H**G 72	136 NM**S** 138	165 AAG**N** 168	248 T**S** 249	615	[21]
23	Serine protease	3TI9_A	40 V**D**TG 43	105 **H**G 106	174 NM**S** 176	204 AAG**N** 207	276 T**S** 277	500	[22]
24	Basic extracellular subtilisin-like protease BprV	3TI7_A	40 V**D**TG 43	105 **H**G 106	174 NM**S** 176	204 AAG**N** 207	276 T**S** 277	394	[22]
25	Serine protease	5YL7_A	184 L**D**TG 187	244 **H**G 245	313 NM**S** 315	343 AAG**N** 346	424 T**S** 425	1055	[23]
26	Aqualysin-1	4DZT_A	38 I**D**TG 41	70 **H**G 71	126 NM**S** 128	154 AAG**N** 157	221 T**S** 222	1011	[24]
27	Protease	5Z6O_A	41 V**D**TG 44	73 **H**G 74	134 NM**S** 136	162 AAG**N** 165	227 T**S** 228	446	[25]
28	Subtilase family protein	4KG7_A	94 I**D**TG 97	126 **H**G 127	199 NI**S** 201	235 AAG**N** 238	340 T**S** 341	605	[26]
Additional members (Asn group)									
29	Extracellular serine protease	3HJR_A	77 V**D**DG 80	115 **H**G 116	175 NQ**S** 177	215 AAG**N** 218	335 T**S** 336	740	[27]
30	Nisin leader peptide-processing serine protease NisP	4MZD_A	258 I**D**SG 261	306 **H**G 307	361 NI**S** 363	404 ALG**N** 407	511 N**S** 512	702	[28]

31	Protease CspB	4I0W_B	125 L**D**TG 128	183 **H**G 184	250 NI**S** 252	284 AAG**N** 287	493 T**S** 494	710	[29]
32	Subtilisin-like serine protease	3AFG_A	146 I**D**TG 149	180 **H**G 181	245 NL**S** 247	277 AAG**N** 280	358 T**A** 359	633	[30]
33	C5a peptidase	3EIF_A	129 I**D**AG 132	193 **H**G 194	258 NM**S** 260	292 SAG**N** 295	511 T**S** 512	1050	[31]
				Ser/Thr group					
34	Putative 36kDa protease	2IXT_A	33 L**D**TG 36	71 **H**G 72	137 SM**S** 139	166 AAG**N** 169	249 T**S** 250	2271<br>2078	[21]
35	Proteinase K	2PWA_A	38 I**D**TG 41	69 **H**G 70	130 SL**S** 132	158 AAG**N** 161	223 T**S** 224	5004<br>5003	[32]
36	Tk-subtilisin	2Z30_A	114 L**D**TG 117	153 **H**G 154	232 SM**S** 234	261 ASG**N** 264	323 T**A** 324	1026<br>1016	[33]
37	Intracellular subtilisin protease	2X8J_F	48 I**D**TG 51	86 **H**G 87	151 TM**S** 153	180 AAG**N** 183	249 T**A** 250	2166<br>2065	[34]
38	Furin precursor	1P8J_A	152 L**D**DG 155	194 **H**G 195	251 SA**S** 253	292 ASG**N** 295	367 T**S** 368	4170<br>4074	[35]
39	Kexin	2ID4_A	174 V**D**DG 177	213 **H**G 214	270 SC**S** 272	311 ASG**N** 314	384 T**S** 385	987<br>937	[36]
40	Alkaline serine protease	1V6C_A	29 I**D**SG 32	65 **H**G 66	127 TM**S** 129	156 AAG**N** 159	368 T**S** 369	524<br>529	[37]
41	Thermitase	1THM_A	37 V**D**TG 40	71 **H**G 72	131 SL**S** 133	160 AAG**N** 163	224 T**S** 225	312<br>316	[38]
42	Intracellular serine protease (Fragment)	7Y6M_C	65 L**D**TG 68	103 **H**G 104	163 SM**S** 165	192 AAG**N** 195	261 T**S** 262	N/A[c]<br>N/A	[39]
43	Protease	1WMD_A	29 A**D**TG 32	68 **H**G 69	127 TN**S** 129	159 AAG**N** 162	254 T**S** 255	3020<br>3018	[40]
44	Serine protease	6F9M_A	48 L**D**TG 51	86 **H**G 87	152 SM**S** 154	181 AAG**N** 184	250 T**S** 251	538<br>560	[41]
45	Alkaline serine protease ver112	3F7M_A	142 I**D**TG 145	171 **H**G 172	234 SM**S** 236	262 AAG**N** 265	327 T**S** 328	385<br>466	[12]
				Additional members (Ser/Thr group)					
46	Cucumisin	4YN3_A	139 L**D**TG 142	204 **H**G 205	271 SL**S** 273	304 SAG**N** 307	524 T**S** 525	974<br>1085	[42]
47	Enzyme subtilase SubHal	5FBZ_A	29 A**D**TG 32	68 **H**G 69	126 TN**S** 128	158 AAG**N** 161	253 T**S** 254	840<br>736	[19]
				Xaa group					
48	Proprotein convertase subtilisin/kexin type 9	6U26_B	185 L**D**TS 188	226 **H**G 227	286 LL**P** 288	314 AAG**N** 317	385 T**S** 386	N/A<br>N/A	[43]
49	Thiazoline oxidase/ subtilisin-like protease	4H6X_A	547 I**D**GD 550	618 **H**A 619	681 HC**A** 683	714 PTG**N** 717	782 T**S** 783	N/A<br>947	[6]

N	Protein	PDB ID	Dipeptide	BaseAcid	CHO	Oxy	Nuc	HOH	Ref.
				Family: Serine-carboxyl proteinase, SCP[d]					
				Asn group					
50	Serine-carboxyl proteinase	1GA6_A	33 IT-- 34	80 **E**WDL**D** 84	131 NV**S** 133	167 SSG**D** 170	286 T**S** 287	403	[44]
				Ser/Thr group					
51	Kumamolysin	1GT9_2	31 IE-- 32	78 **E**VEL**D** 82	126 SI**S** 128	161 AAG**D** 164	277 T**S** 278	2120<br>2036	[45]
52	Kumamolisin-As	1SIO_A	31 IE-- 32	78 **E**VEL**D** 82	126 SI**S** 128	161 AAG**D** 164	277 T**S** 278	1505<br>1502	[46]
				Additional members (Ser/Thr group)					
53	Tripeptidyl-peptidase 1	3EDY_A	228 AQ--229	272 **E**ASL**D** 276	322 TV**S** 324	357 ASG**D** 360	474 T**S** 475	715<br>807	[47]

[a] Five main catalytic residues are shown in bold.

[b] The "HOH" column shows PDB numbers of the HOH_{I} and HOH_{II} water molecules. If only one number is given, it belongs to the HOH_{II} water molecule. If two numbers are given, the upper number refers to HOH_{I}, and the lower number refers to HOH_{II}.

[c] N/A - Not Available.
[d] The designation "--" is used only to emphasize the presence dipeptide without a catalytically important amino acid in the SCP family compared to the Acid tetrapeptide in the subtilases family.

References

1. Kuhn P, Knapp M, Soltis SM, Ganshaw G, Thoene M, Bott R. The 0.78 A structure of a serine protease: Bacillus lentus subtilisin. Biochemistry. 1998 Sep 29;37(39):13446-52. doi: 10.1021/bi9813983. PMID: 9753430.
2. Radisky ES, Kwan G, Karen Lu CJ, Koshland DE Jr. Binding, proteolytic, and crystallographic analyses of mutations at the protease-inhibitor interface of the subtilisin BPN'/chymotrypsin inhibitor 2 complex. Biochemistry. 2004 Nov 2;43(43):13648-56. doi: 10.1021/bi048797k. PMID: 15504027.
3. Horn JR, Ramaswamy S, Murphy KP. Structure and energetics of protein-protein interactions: the role of conformational heterogeneity in OMTKY3 binding to serine proteases. J Mol Biol. 2003 Aug 8;331(2):497-508. doi: 10.1016/s0022-2836(03)00783-6. PMID: 12888355.
4. Jain SC, Shinde U, Li Y, Inouye M, Berman HM. The crystal structure of an autoprocessed Ser221Cys-subtilisin E-propeptide complex at 2.0 A resolution. J Mol Biol. 1998 Nov 20;284(1):137-44. doi: 10.1006/jmbi.1998.2161. PMID: 9811547.
5. Arnórsdóttir J, Kristjánsson MM, Ficner R. Crystal structure of a subtilisin-like serine proteinase from a psychrotrophic Vibrio species reveals structural aspects of cold adaptation. FEBS J. 2005 Feb;272(3):832-45. doi: 10.1111/j.1742-4658.2005.04523.x. PMID: 15670163.
6. Agarwal V, Pierce E, McIntosh J, Schmidt EW, Nair SK. Structures of cyanobactin maturation enzymes define a family of transamidating proteases. Chem Biol. 2012 Nov 21;19(11):1411-22. doi: 10.1016/j.chembiol.2012.09.012. PMID: 23177196; PMCID: PMC10294700.
7. Wu WL, Chen MY, Tu IF, Lin YC, EswarKumar N, Chen MY, Ho MC, Wu SH. The discovery of novel heat-stable keratinases from Meiothermus taiwanensis WR-220 and other extremophiles. Sci Rep. 2017 Jul 5;7(1):4658. doi: 10.1038/s41598-017-04723-4. PMID: 28680127; PMCID: PMC5498600.
8. Shenoy RT, Thangamani S, Velazquez-Campoy A, Ho B, Ding JL, Sivaraman J. Structural basis for dual-inhibition mechanism of a non-classical Kazal-type serine protease inhibitor from horseshoe crab in complex with subtilisin. PLoS One. 2011 Apr 26;6(4):e18838. doi: 10.1371/journal.pone.0018838. PMID: 21541315; PMCID: PMC3082530.
9. Derache C, Epinette C, Roussel A, Gabant G, Cadene M, Korkmaz B, Gauthier F, Kellenberger C. Crystal structure of greglin, a novel non-classical Kazal inhibitor, in complex with subtilisin. FEBS J. 2012 Dec;279(24):4466-78. doi: 10.1111/febs.12033. Epub 2012 Nov 12. PMID: 23075397.
10. Shirai T, Suzuki A, Yamane T, Ashida T, Kobayashi T, Hitomi J, Ito S. High-resolution crystal structure of M-protease: phylogeny aided analysis of the high-alkaline adaptation mechanism. Protein Eng. 1997 Jun;10(6):627-34. doi: 10.1093/protein/10.6.627. PMID: 9278275.
11. Kennan RM, Wong W, Dhungyel OP, Han X, Wong D, Parker D, Rosado CJ, Law RH, McGowan S, Reeve SB, Levina V, Powers GA, Pike RN, Bottomley SP, Smith AI, Marsh I, Whittington RJ, Whisstock JC, Porter CJ, Rood JI. The subtilisin-like protease AprV2 is required for virulence and uses a novel disulphide-tethered exosite to bind substrates. PLoS Pathog. 2010 Nov 24;6(11):e1001210. doi: 10.1371/journal.ppat.1001210. PMID: 21124876; PMCID: PMC2991261.
12. Liang L, Meng Z, Ye F, Yang J, Liu S, Sun Y, Guo Y, Mi Q, Huang X, Zou C, Rao Z, Lou Z, Zhang KQ. The crystal structures of two cuticle-degrading proteases from nematophagous fungi and their contribution to infection against nematodes. FASEB J. 2010 May;24(5):1391-400. doi: 10.1096/fj.09-136408. Epub 2009 Dec 9. PMID: 20007510.
13. Withers-Martinez C, Strath M, Hackett F, Haire LF, Howell SA, Walker PA, Christodoulou E, Dodson GG, Blackman MJ. The malaria parasite egress protease SUB1 is a calcium-dependent redox switch subtilisin. Nat Commun. 2014 May 2;5:3726. doi: 10.1038/ncomms4726. Erratum in: Nat Commun. 2014;5:4031. Evangelos, Christodoulou [corrected to Christodoulou, Evangelos]. PMID: 24785947; PMCID: PMC4024747.
14. Kim JS, Kluskens LD, de Vos WM, Huber R, van der Oost J. Crystal structure of fervidolysin from Fervidobacterium pennivorans, a keratinolytic enzyme related to subtilisin. J Mol Biol. 2004 Jan 16;335(3):787-

97. doi: 10.1016/j.jmb.2003.11.006. PMID: 14687574.
15. Dauter Z, Betzel C, Genov N, Pipon N, Wilson KS. Complex between the subtilisin from a mesophilic bacterium and the leech inhibitor eglin-C. Acta Crystallogr B. 1991 Oct 1;47 (Pt 5):707-30. doi: 10.1107/s0108768191004202. PMID: 1793542.
16. Smith CA, Toogood HS, Baker HM, Daniel RM, Baker EN. Calcium-mediated thermostability in the subtilisin superfamily: the crystal structure of Bacillus Ak.1 protease at 1.8 A resolution. J Mol Biol. 1999 Dec 10;294(4):1027-40. doi: 10.1006/jmbi.1999.3291. PMID: 10588904.
17. Eschenburg S, Genov N, Peters K, Fittkau S, Stoeva S, Wilson KS, Betzel C. Crystal structure of subtilisin DY, a random mutant of subtilisin Carlsberg. Eur J Biochem. 1998 Oct 15;257(2):309-18. doi: 10.1046/j.1432-1327.1998.2570309.x. PMID: 9826175.
18. Goddette DW, Paech C, Yang SS, Mielenz JR, Bystroff C, Wilke ME, Fletterick RJ. The crystal structure of the Bacillus lentus alkaline protease, subtilisin BL, at 1.4 A resolution. J Mol Biol. 1992 Nov 20;228(2):580-95. doi: 10.1016/0022-2836(92)90843-9. PMID: 1453465.
19. Dohnalek J, McAuley KE, Brzozowski AM, Østergaard PR, Svendsen A, Wilson KS. Stabilization of Enzymes by Metal Binding: Structures of Two Alkalophilic Bacillus Subtilases and Analysis of the Second Metal-Binding Site of the Subtilase Family. Chapter in Understanding enzymes; Function, Design, Engineering and Analysis, Pan Stanford Publishing, ed. A. Svendsen, 2016, 203-266, ISBN 789814669320, doi: 10.4032/9789814669337.
20. Helland R, Larsen AN, Smalås AO, Willassen NP. The 1.8 A crystal structure of a proteinase K-like enzyme from a psychrotroph Serratia species. FEBS J. 2006 Jan;273(1):61-71. doi: 10.1111/j.1742-4658.2005.05040.x. PMID: 16367748.
21. Almog O, González A, Godin N, de Leeuw M, Mekel MJ, Klein D, Braun S, Shoham G, Walter RL. The crystal structures of the psychrophilic subtilisin S41 and the mesophilic subtilisin Sph reveal the same calcium-loaded state. Proteins. 2009 Feb 1;74(2):489-96. doi: 10.1002/prot.22175. PMID: 18655058.
22. Wong W, Wijeyewickrema LC, Kennan RM, Reeve SB, Steer DL, Reboul C, Smith AI, Pike RN, Rood JI, Whisstock JC, Porter CJ. S1 pocket of a bacterially derived subtilisin-like protease underpins effective tissue destruction. J Biol Chem. 2011 Dec 9;286(49):42180-42187. doi: 10.1074/jbc.M111.298711. Epub 2011 Oct 11. PMID: 21990366; PMCID: PMC3234984.
23. Park HJ, Lee CW, Kim D, Do H, Han SJ, Kim JE, Koo BH, Lee JH, Yim JH. Crystal structure of a cold-active protease (Pro21717) from the psychrophilic bacterium, Pseudoalteromonas arctica PAMC 21717, at 1.4 Å resolution: Structural adaptations to cold and functional analysis of a laundry detergent enzyme. PLoS One. 2018 Feb 21;13(2):e0191740. doi: 10.1371/journal.pone.0191740. PMID: 29466378; PMCID: PMC5821440.
24. Barnett BL, Green PR, Strickland LC, Oliver JD, Rydel T, Sullivan JF. Aqualysin I: the crystal structure of a serine protease from an extreme thermophile, Thermus aquaticus YT-1. 2012. doi: 10.2210/pdb4DZT/pdb.
25. Koszelak S, Ng JD, Day J, Ko TP, Greenwood A, McPherson A. The crystallographic structure of the subtilisin protease from Penicillium cyclopium. Biochemistry. 1997 Jun 3;36(22):6597-604. doi: 10.1021/bi963189t. PMID: 9184139.
26. Wagner JM, Evans TJ, Chen J, Zhu H, Houben EN, Bitter W, Korotkov KV. Understanding specificity of the mycosin proteases in ESX/type VII secretion by structural and functional analysis. J Struct Biol. 2013 Nov;184(2):115-28. doi: 10.1016/j.jsb.2013.09.022. Epub 2013 Oct 7. PMID: 24113528; PMCID: PMC3859186.
27. Kobayashi H, Utsunomiya H, Yamanaka H, Sei Y, Katunuma N, Okamoto K, Tsuge H. Structural basis for the kexin-like serine protease from Aeromonas sobria as sepsis-causing factor. J Biol Chem. 2009 Oct 2;284(40):27655-63. doi: 10.1074/jbc.M109.006114. Epub 2009 Aug 4. PMID: 19654332; PMCID: PMC2785694.
28. Xu Y, Li X, Li R, Li S, Ni H, Wang H, Xu H, Zhou W, Saris PE, Yang W, Qiao M, Rao Z. Structure of the nisin leader peptidase NisP revealing a C-terminal autocleavage activity. Acta Crystallogr D Biol Crystallogr. 2014 Jun;70(Pt 6):1499-505. doi: 10.1107/S1399004714004234. Epub 2014 May 23. PMID: 24914961.
29. Adams CM, Eckenroth BE, Putnam EE, Doublié S, Shen A. Structural and functional analysis of the CspB protease required for Clostridium spore germination. PLoS Pathog. 2013 Feb;9(2):e1003165. doi: 10.1371/journal.ppat.1003165. Epub 2013 Feb 7. PMID: 23408892; PMCID: PMC3567191.
30. Foophow T, Tanaka S, Angkawidjaja C, Koga Y, Takano K, Kanaya S. Crystal structure of a subtilisin homologue, Tk-SP, from Thermococcus kodakaraensis: requirement of a C-terminal beta-jelly roll domain for hyperstability. J Mol Biol. 2010 Jul 23;400(4):865-77. doi: 10.1016/j.jmb.2010.05.064. Epub 2010 Jun 1. PMID: 20595040.

31. Kagawa TF, O'Connell MR, Mouat P, Paoli M, O'Toole PW, Cooney JC. Model for substrate interactions in C5a peptidase from Streptococcus pyogenes: A 1.9 A crystal structure of the active form of ScpA. J Mol Biol. 2009 Feb 27;386(3):754-72. doi: 10.1016/j.jmb.2008.12.074. Epub 2009 Jan 6. PMID: 19152799.
32. Jain R, Singh N, Perbandt M, Betzel C, Sharma S, Kaur P, Srinivasan A, Singh TP. Crystal structure of the complex of Proteinase K with Alanine Boronic Acid at 0.83A Resolution. 2007. doi: 10.2210/pdb2PWA/pdb.
33. Tanaka SI, Matsumura H, Koga Y, Takano K, Kanaya S. Four new crystal structures of Tk-subtilisin in unautoprocessed, autoprocessed and mature forms: insight into structural changes during maturation. J Mol Biol. 2007 Sep 28;372(4):1055-1069. doi: 10.1016/j.jmb.2007.07.027. Epub 2007 Jul 26. PMID: 17706669.
34. Vévodová J, Gamble M, Künze G, Ariza A, Dodson E, Jones DD, Wilson KS. Crystal structure of an intracellular subtilisin reveals novel structural features unique to this subtilisin family. Structure. 2010 Jun 9;18(6):744-55. doi: 10.1016/j.str.2010.03.008. PMID: 20541512.
35. Henrich S, Cameron A, Bourenkov GP, Kiefersauer R, Huber R, Lindberg I, Bode W, Than ME. The crystal structure of the proprotein processing proteinase furin explains its stringent specificity. Nat Struct Biol. 2003 Jul;10(7):520-6. doi: 10.1038/nsb941. Erratum in: Nat Struct Biol. 2003 Aug;10(8):669. PMID: 12794637.
36. Wheatley JL, Holyoak T. Differential P1 arginine and lysine recognition in the prototypical proprotein convertase Kex2. Proc Natl Acad Sci U S A. 2007 Apr 17;104(16):6626-31. doi: 10.1073/pnas.0701983104. Epub 2007 Apr 10. PMID: 17426142; PMCID: PMC1871836.
37. Dong D, Ihara T, Motoshima H, Watanabe K. Crystal Structure of Psychrophilic Subtilisin-like Protease Apa1 from Antarctic Psychrotroph Pseudoalteromonas sp. AS-11. 2004. doi: 10.2210/pdb1V6C/pdb.
38. Teplyakov AV, Kuranova IP, Harutyunyan EH, Vainshtein BK, Frömmel C, Höhne WE, Wilson KS. Crystal structure of thermitase at 1.4 A resolution. J Mol Biol. 1990 Jul 5;214(1):261-79. doi: 10.1016/0022-2836(90)90160-n. PMID: 2196375.
39. Hussin N, Jamaluddin H, Jonet MA. Intracellular Subtilisin from Bacillus sp. 2022. doi: 10.2210/pdb7Y6M/pdb.
40. Nonaka T, Fujihashi M, Kita A, Saeki K, Ito S, Horikoshi K, Miki K. The crystal structure of an oxidatively stable subtilisin-like alkaline serine protease, KP-43, with a C-terminal beta-barrel domain. J Biol Chem. 2004 Nov 5;279(45):47344-51. doi: 10.1074/jbc.M409089200. Epub 2004 Sep 1. PMID: 15342641.
41. Bjerga GEK, Larsen Ø, Arsın H, Williamson A, García-Moyano A, Leiros I, Puntervoll P. Mutational analysis of the pro-peptide of a marine intracellular subtilisin protease supports its role in inhibition. Proteins. 2018 Sep;86(9):965-977. doi: 10.1002/prot.25528. Epub 2018 Sep 17. PMID: 29907987; PMCID: PMC6220982.
42. Sotokawauchi A, Kato-Murayama M, Murayama K, Hosaka T, Maeda I, Onjo M, Ohsawa N, Kato DI, Arima K, Shirouzu M. Structural basis of cucumisin protease activity regulation by its propeptide. J Biochem. 2017 Jan;161(1):45-53. doi: 10.1093/jb/mvw053. Epub 2016 Sep 10. PMID: 27616715.
43. Petrilli WL, Adam GC, Erdmann RS, Abeywickrema P, Agnani V, Ai X, Baysarowich J, Byrne N, Caldwell JP, Chang W, DiNunzio E, Feng Z, Ford R, Ha S, Huang Y, Hubbard B, Johnston JM, Kavana M, Lisnock JM, Liang R, Lu J, Lu Z, Meng J, Orth P, Palyha O, Parthasarathy G, Salowe SP, Sharma S, Shipman J, Soisson SM, Strack AM, Youm H, Zhao K, Zink DL, Zokian H, Addona GH, Akinsanya K, Tata JR, Xiong Y, Imbriglio JE. From Screening to Targeted Degradation: Strategies for the Discovery and Optimization of Small Molecule Ligands for PCSK9. Cell Chem Biol. 2020 Jan 16;27(1):32-40.e3. doi: 10.1016/j.chembiol.2019.10.002. Epub 2019 Oct 22. Erratum in: Cell Chem Biol. 2021 Feb 18;28(2):243. doi: 10.1016/j.chembiol.2021.01.019. PMID: 31653597.
44. Wlodawer A, Li M, Dauter Z, Gustchina A, Uchida K, Oyama H, Dunn BM, Oda K. Carboxyl proteinase from Pseudomonas defines a novel family of subtilisin-like enzymes. Nat Struct Biol. 2001 May;8(5):442-6. doi: 10.1038/87610. PMID: 11323721.
45. Comellas-Bigler M, Fuentes-Prior P, Maskos K, Huber R, Oyama H, Uchida K, Dunn BM, Oda K, Bode W. The 1.4 a crystal structure of kumamolysin: a thermostable serine-carboxyl-type proteinase. Structure. 2002 Jun;10(6):865-76. doi: 10.1016/s0969-2126(02)00772-4. PMID: 12057200.
46. Wlodawer A, Li M, Gustchina A, Tsuruoka N, Ashida M, Minakata H, Oyama H, Oda K, Nishino T, Nakayama T. Crystallographic and biochemical investigations of kumamolisin-As, a serine-carboxyl peptidase with collagenase activity. J Biol Chem. 2004 May 14;279(20):21500-10. doi: 10.1074/jbc.M401141200. Epub 2004 Mar 10. PMID: 15014068.
47. Guhaniyogi J, Sohar I, Das K, Stock AM, Lobel P. Crystal structure and autoactivation pathway of the precursor form of human tripeptidyl-peptidase 1, the enzyme deficient in late infantile ceroid lipofuscinosis. J Biol Chem.

2009 Feb 6;284(6):3985-97. doi: 10.1074/jbc.M806943200. Epub 2008 Nov 26. PMID: 19038967; PMCID: PMC2635056.
